# Mobile Stroke Unit Operational Metrics: Institutional Experience, Systematic Review and Meta-Analysis

**DOI:** 10.3389/fneur.2022.868051

**Published:** 2022-05-09

**Authors:** Nathaniel R. Ellens, Derrek Schartz, Redi Rahmani, Sajal Medha K. Akkipeddi, Adam G. Kelly, Curtis G. Benesch, Stephanie A. Parker, Jason L. Burgett, Diana Proper, Webster H. Pilcher, Thomas K. Mattingly, James C. Grotta, Tarun Bhalla, Matthew T. Bender

**Affiliations:** ^1^Department of Neurosurgery, University of Rochester Medical Center, Rochester, NY, United States; ^2^Department of Imaging Sciences, University of Rochester Medical Center, Rochester, NY, United States; ^3^Department of Neurology, University of Rochester Medical Center, Rochester, NY, United States; ^4^Department of Neurology, University of Texas McGovern Medical School, Houston, TX, United States; ^5^Mobile Stroke Unit, Memorial Hermann Hospital—Texas Medical Center, Houston, TX, United States

**Keywords:** mobile stroke unit (MSU), ambulance, mechanical thrombectomy (MT), tissue plasminogen activator (tPA), operational performance

## Abstract

**Background:**

The available literature on mobile stroke units (MSU) has focused on clinical outcomes, rather than operational performance. Our objective was to establish normalized metrics and to conduct a meta-analysis of the current literature on MSU performance.

**Methods:**

Our MSU in upstate New York serves 741,000 people. We present prospectively collected, retrospectively analyzed data from the inception of our MSU in October of 2018, through March of 2021. Rates of transportation/dispatch and MSU utilization were reported. We also performed a meta-analysis using MEDLINE, SCOPUS, and Cochrane Library databases, calculating rates of tPA/dispatch, tPA-per-24-operational-hours (“per day”), mechanical thrombectomy (MT)/dispatch and MT/day.

**Results:**

Our MSU was dispatched 1,719 times in 606 days (8.5 dispatches/24-operational-hours) and transported 324 patients (18.8%) to the hospital. Intravenous tPA was administered in 64 patients (3.7% of dispatches) and the rate of tPA/day was 0.317 (95% CI 0.150–0.567). MT was performed in 24 patients (1.4% of dispatches) for a MT/day rate of 0.119 (95% CI 0.074–0.163). The MSU was in use for 38,742 minutes out of 290,760 total available minutes (13.3% utilization rate). Our meta-analysis included 14 articles. Eight studies were included in the analysis of tPA/dispatch (342/5,862) for a rate of 7.2% (95% CI 4.8–9.5%, I^2^ = 92%) and 11 were included in the analysis of tPA/day (1,858/4,961) for a rate of 0.358 (95% CI 0.215–0.502, I^2^ = 99%). Seven studies were included for MT/dispatch (102/5,335) for a rate of 2.0% (95% CI 1.2–2.8%, I^2^ = 67%) and MT/day (103/1,249) for a rate of 0.092 (95% CI 0.046–0.138, I^2^ = 91%).

**Conclusions:**

In this single institution retrospective study and meta-analysis, we outline the following operational metrics: tPA/dispatch, tPA/day, MT/dispatch, MT/day, and utilization rate. These metrics are useful for internal and external comparison for institutions with or considering developing mobile stroke programs.

## Introduction

Since mobile stroke units (MSU) were first described in 2003 in Germany, numerous studies have shown MSU care expedites intravenous thrombolysis and mechanical thrombectomy compared to standard emergency medical services (1–7). Recently, two large, prospective controlled trials have shown improved clinical outcomes 90 days after presentation with acute ischemic stroke in patients receiving MSU care as compared to traditional emergency medical services (8, 9). These compelling data have raised the question, “Does My District Need a Mobile Stroke Unit?” (10).

Because MSU operations require significant personnel and material resources, cost-effectiveness and viability will vary with local circumstances (11). The decision to establish a mobile stroke unit must be made in consideration of local case volume, geography, and infrastructure. The purpose of this manuscript was to establish standard metrics for reporting MSU operational efficiency and to benchmark those numbers using our institutional experience and a meta-analysis of the current literature.

## Methods

### Retrospective Single Center Cohort Analysis

The authors performed a retrospective analysis of a prospective database of stroke patients at the University of Rochester, from October 2018 through March 2021. At our institution, the mobile stroke unit services a population of ~741,770 people in the greater Rochester area. The MSU is available on weekdays for 8 h per day and is staffed by a registered nurse and a CT technician, each with specialized training, along with a paramedic and emergency medical technician (EMT) (12). Our MSU is dispatched along with a separate emergency medical service (EMS) unit for all 911 calls identified as suspected stroke (Card 28) as defined by the Medical Priority Dispatch System (version 13.1, International Academies of Emergency Dispatch, Salt Lake City, UT). Accuracy of code 28 dispatches in our catchment is consistent with national standards with approximately one-third representing patients ultimately diagnosed with transient ischemia or stroke. Our MSU can be dispatched by first responder request but is not permitted to self-attach after hearing of possible stroke over EMS communications. The MSU works with an on-call teleneurologist. Each eligible patient undergoes a formal evaluation on-scene using a National Institutes of Health Stroke Scale (NIHSS), followed by non-contrast head CT. A decision is then made regarding immediate tPA administration and, when clinically indicated, patients are transferred to a comprehensive stroke center for mechanical thrombectomy.

Institutional review board approval was obtained to collect and report the data at our institution. Operational metrics were collected and included dispatches, transport, tPA administration, mechanical thrombectomy (MT), and operational hours in service. Utilization was calculated by dividing the duration of time from dispatch to return to service for all MSU dispatches, by the total time the MSU was available. The authors also performed a systematic review and meta-analysis to determine the operational metrics reported in the literature for MSUs. This was performed up to May of 2021, according to PRISMA (Preferred Reporting Items for Systematic Reviews and Meta-Analysis) guidelines.

### Meta-Analysis Eligibility Criteria and Study Selection

All studies that were identified and analyzed fit the following criteria: the paper was written in English and reported operational metrics of a mobile stroke unit that consisted of dispatches, transports, intravenous tPA administration, MT, and operational hours in service. We included studies of a prospective and retrospective nature, in addition to large case series. Case reports were excluded. Local, regional, and international studies were included. Data was abstracted from publications and/or supplementary data in all cases except BEST-MSU, which consists of 7 sites (8). BEST-MSU investigators provided details regarding launch dates, operational hours, and site-specific enrollment to enable accurate determination of pooled tPA/day.

### Information Sources, Literature Search, and Data Collection

**Figure 1:**
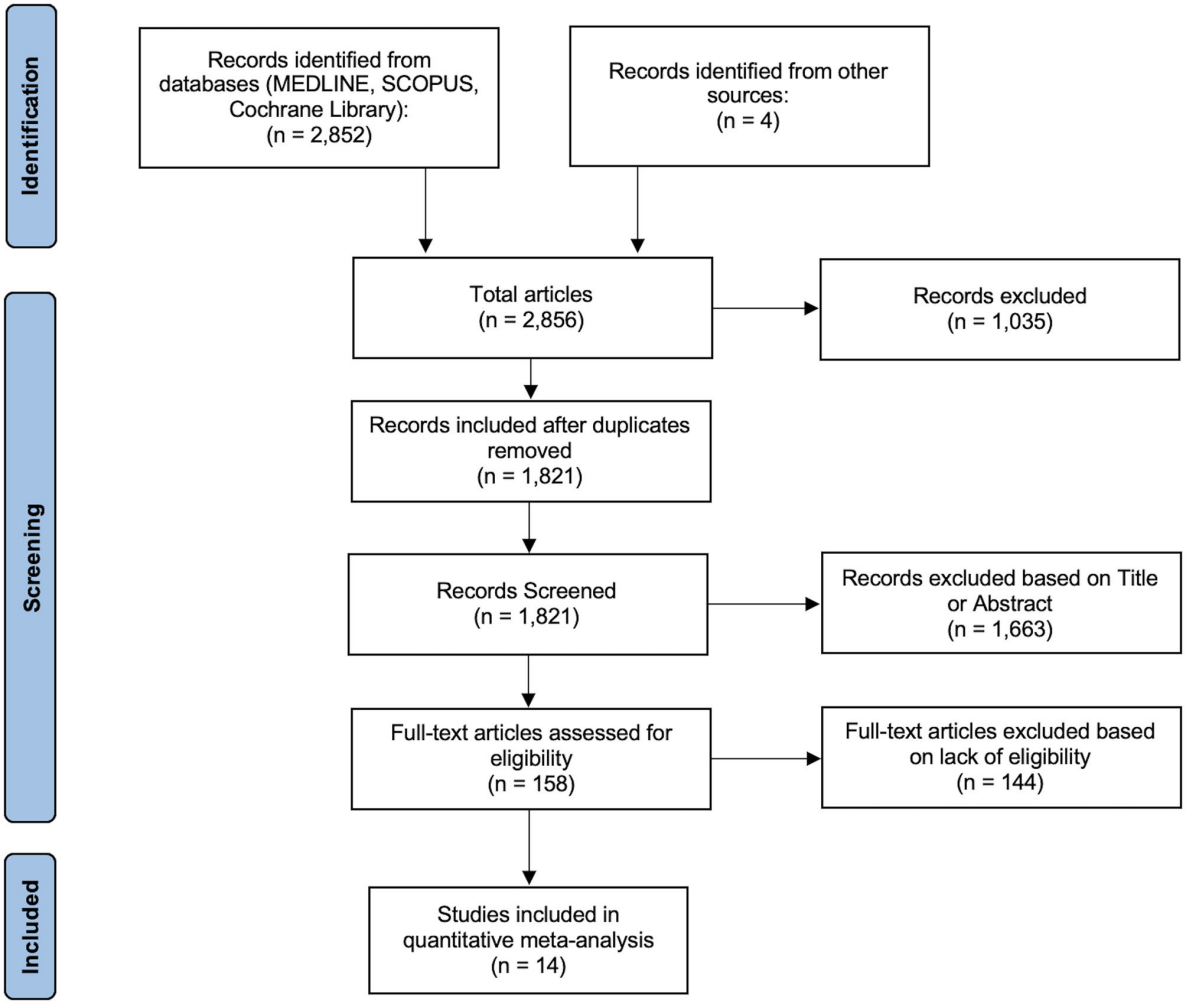
PRISMA flow diagram. This diagram demonstrates the systematic method used for the identification, screening, and inclusion of articles that met criteria for inclusion within the meta-analysis.

Relevant studies were identified by searching the MEDLINE, SCOPUS and Cochrane Library databases up to May of 2021 using the following terms: (mobile stroke unit), (mobile stroke) AND (efficiency), (mobile stroke program), (mobile stroke) AND (dispatch), (mobile stroke) AND (utilization). Any duplicates that were identified within this search were then removed (Figure 1). The initial screening process was performed by reading the article title, followed by the abstract. If there was still uncertainty regarding the article's relevance, the full article manuscript was read. Two independent reviewers rigorously reviewed all final manuscripts to confirm their relevance and the decision for inclusion. Any cases of disagreement were resolved by consensus with the remainder of the authors. All studies that passed the above criteria were included within this meta-analysis.

### Results Synthesis and Statistical Analysis

Forest plots depicting the pooled proportion for the transportations/dispatch, tPA/dispatch, tPA/day, MT/dispatch, and MT/day analyses were generated along with 95% confidence intervals (CI) using a random effects model. “Per day” is used to mean “per 24 operational hours” and reflects the fact that individual programs run different shift lengths. Pooled, meta-analytic rates were calculated from weighted proportions of the included studies and are not derived simply from the composite rates. Individual studies were weighted by the inverse variance of their estimated variances to account for inter-study variation (DerSimion Laird). All forest plots and corresponding statistical analysis, were generated using the OpenMeta[Analyst] from Brown University (http://www.cebm.brown.edu/openmeta/).

Statistical heterogeneity was assessed using Cochran's Q statistic and described using the I^2^ measure. An I^2^ of 50 and 75% were used as benchmarks for moderate and high heterogeneity among the included studies, respectively. A *P* value of under 0.05 was used as a benchmark for significance within the heterogeneity assessment. Assessment of potential publication bias was also completed for each analysis using funnel plots and Egger's regression test, implemented with MedCalc statistical software (https://www.medcalc.org). A *P* > 0.05 was used to indicate non-significant bias.

## Results

### Retrospective Single Center Cohort Analysis

The MSU at our institution was dispatched 1,719 times over a period of 606 days for an average rate of 8.5 dispatches/24-hour-day. Of these 1,719 dispatches, 324 patients (18.8%) were transported to a nearby hospital. Systemic intravenous tPA was administered in 64 patients (3.7% of all dispatches) and the rate of tPA administration per day was 0.317 (95% CI 0.150–0.567). Twenty-four patients were found to have large vessel occlusions and underwent mechanical thrombectomy (1.4% of dispatches) for an MT/day rate of 0.119 (95% CI 0.074–0.163). Of the 290,760 total minutes the MSU was available, it was in use for 38,742 mins (13.3% utilization rate).

**Figure 2:**
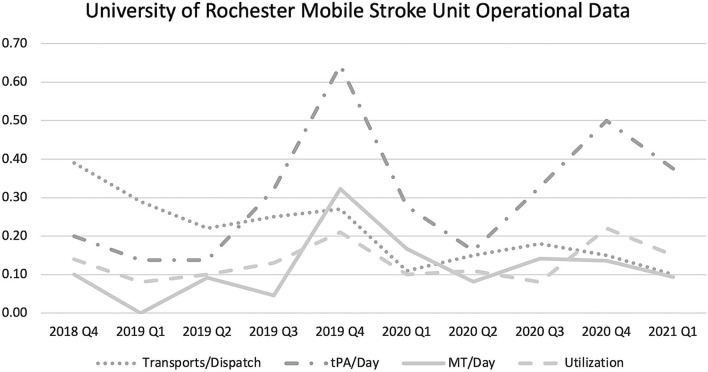
University of Rochester Medical Center Mobile Stroke Unit Operational Data. The data shown here outlines the mobile stroke unit experience at the University of Rochester from its inception in October of 2018, through March of 2021. tPA, tissue plasminogen activator; MT, mechanical thrombectomy.

Our MSU launched in October of 2018, serving the downtown Rochester population of 206,284. For the first 3 quarters, we noted a tPA/day rate of 0.159 and an MT/day rate of 0.064. We then expanded to serve the greater Rochester area in June of 2019, increasing our catchment population to 741,770 for quarters 4 through 10. Our tPA/day and MT/day rates increased to 0.373 and 0.141, following this expansion. As our institution gained experience, we also noticed a trend of increasing dispatches/24-h-day, with a rate of 6.4 during the first five quarters, and a rate of 10.7 during the second five quarters. There was a mild decrease in rates of tPA/dispatch (4.2% decreased to 3.4%) and MT/dispatch (1.5 to 1.4%). Average utilization rates remained relatively stable (13%) between the first and second half of our study experience (Figure 2).

### Systematic Literature Search

Using the MEDLINE, SCOPUS, and Cochrane databases we identified 2,856 total articles, which consisted of 1,821 unique articles after removing duplicates. 1,663 articles were then removed after reading the titles and abstracts, leaving a total of 158 articles. The full text of each article was read and 14 of these articles fit the criteria and were included in the analysis. Each of these articles were reviewed independently by two authors (NE and MB) to confirm that they fit the inclusion criteria.

### Meta-Analysis of Proportion

**Figure 3:**
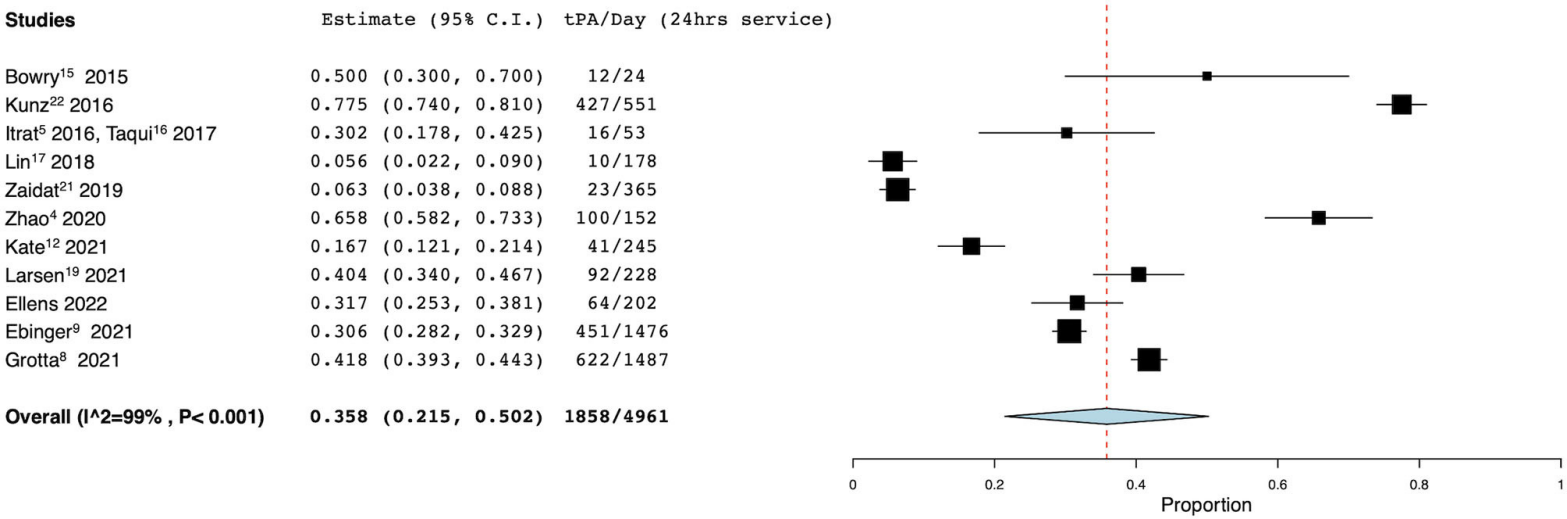
Rate of tPA administration per day. Forest plot demonstrating the results from the meta-analysis of rates of tPA administration per day. tPA, tissue plasminogen activator; CI, confidence interval.

**Figure 4:**
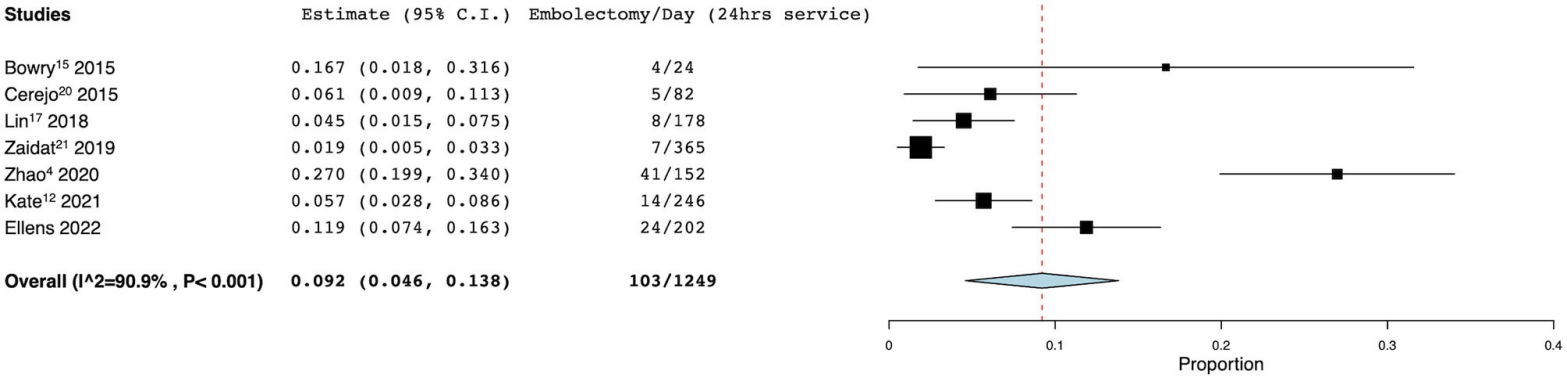
Rate of mechanical thrombectomy per day. Forest plot demonstrating the results from the meta-analysis of the rate of mechanical thrombectomy per day. CI, confidence interval.

There were 8 studies, including the authors' current data, which met the inclusion criteria for pooled analysis of tPA administration per dispatch (4, 5, 13–18). In these studies, there were 5,862 dispatches, of whom 342 were administered tPA for an average rate of 7.2% (95% CI 4.8–9.5%). Seven unique studies met inclusion criteria for analysis of MT/dispatch (4, 13, 14, 16, 17, 19). These seven studies reported a total of 102 patients who underwent mechanical thrombectomy out of a total of 5,335 dispatches. The pooled average rate was 2.0% (95% CI 1.2–2.8%). Eleven studies reported information regarding rates of tPA administration based on time of MSU availability (4, 5, 8, 9, 13–16, 18, 20, 21). Collectively, tPA was administered 1,858 times over 4,961 available days (defined as 24-h period), for a rate of 0.358 (95% CI 0.215–0.502) (Figure 3). Seven studies met the inclusion criteria for pooled analysis of thrombectomy per day (4, 13, 14, 19, 21, 22). In these studies, there were 103 thrombectomies over 1,249 available days, for a rate of 0.092 (95% CI 0.046–0.138) (Figure 4). The authors present the only reported utilization rate in the literature thus far, at 13.3%.

### Assessment of Heterogeneity and Bias

I^2^ values for tPA administration per dispatch and per day were 92 and 99% which both corresponded to *p* < 0.001. I^2^ values for MT/dispatch and MT/day were 67 and 91%, which corresponded to *p* = 0.005 and *p* < 0.001 respectively. Together, this indicated a moderate to high degree of interstudy heterogeneity within each analysis (Supplementary Table 1). Egger's test was also utilized to assess for publication bias, with significant bias observed within the transportations per dispatch analysis (*p* = 0.0097). No significant bias was found within the tPA administration per dispatch (*p* = 0.1976), MT/dispatch analysis (*p* = 0.0817), tPA administration per day (*p* = 0.738), and MT per day (*p* = 0.283) (Supplementary Table 2).

## Discussion

This was a single-institution retrospective observational study of mobile stroke unit operational performance as well as a meta-analysis of the literature on MSU operations. Our MSU was dispatched 8.5 times per 24-h-day and intravenous tPA was administered to 3.7% of dispatches while 1.4% underwent mechanical thrombectomy. Our rates of intravenous tPA administration and thrombectomy per day were 0.317 and 0.119 per day, which were similar to the meta-analysis benchmarks of 0.358 and 0.092 per day, respectively. The data for these metrics was abstracted from existing manuscripts but was not explicitly reported in this format. We introduce the metric “MSU utilization rate,” the percentage of operational minutes in which the MSU was deployed on a call, which was 13.3% at our institution over the study period. Operational performance will be an area of increased focus as mobile stroke units increase in number.

## Thrombolysis Metrics

The first standardized metric used to assess MSU operations was tPA administration per 24 h in service. The PHANTOM-S trial in Berlin reported the highest rate at 0.775, followed by Zhao et al. in Melbourne. Notably, these MSU programs service large populations, with Berlin and Melbourne each servicing populations over 1 million people. We report a tPA administration per day rate of 0.317 (95% CI 0.150–0.567), which is similar to the rate of 0.358 (95% CI 0.150–0.567) observed in the meta-analysis. Not surprisingly, this increased as we expanded our catchment area, as larger catchment areas were noted to typically have higher rates of tPA administration per day (4, 20).

The BEST-MSU group, which contributed 30% of service time to our meta-analysis of tPA/day, illustrates the variability and potential of MSU thrombolysis. The pooled rate across the seven programs included in BEST-MSU was 0.418 tPA/day but ranged from 0.040 in Los Angeles to 1.053 tPA/day in Houston, TX (8). The latter, the oldest program in BEST-MSU which accounted for 25% of BEST-MSU service days, may represent a ceiling of thrombolysis productivity that less mature programs can target. Houston uses several strategies to increase volume and enhance dispatch specificity, including monitoring EMS radio for “active pursuit” opportunities and having first responders add the MSU to high-probability calls not originally identified as a stroke. As a result, Houston is dispatched more often than our MSU in Rochester (13.5 vs. 8.5 times/day) and with a higher thrombolysis yield (10 vs. 3.7% tPA/dispatch). Capacity is preserved by deferring to EMS to transport patients that have obvious exclusions for thrombolysis prior to CT scanning: the fraction of transported patients who received tPA was 60% in Houston as compared with 20% at our program. It is important to acknowledge that BEST-MSU data reflects only those patients who met criteria for enrollment in a controlled trial and excludes a small number of patients who received thrombolysis outside the trial.

Prior to settling on intravenous tPA administration per 24 h in service as a performance metric, we first focused on tPA per MSU dispatch. This figure was 3.7% at our institution and 7.2% in the meta-analysis (range 3.6–26.3%). We came to believe time is a better denominator than dispatches, however, since dispatch criteria vary significantly across programs, often based on urban/rural setting. Kate et al. (13) reported the highest rate of tPA administration per dispatch at 26.3%, while serving rural Alberta, a community with a radius of 250 km. Larsen et al. (18) services rural Norway with an MSU using an air-ambulance and rendezvous model, and reports a tPA/dispatch rate of 12.3%. Given the increased time and resource utilization associated with longer dispatches, rural programs may benefit from more selective MSU dispatch to patients more likely to receive thrombolysis. Conversely, Weinberg et al. (17) demonstrated the lowest rate of tPA administration per dispatch at 3.6%, and they service an area of only 83 square miles in Philadelphia.

### Additional Metrics: Embolectomy and Utilization

A small percentage of MSU dispatches will be candidates for endovascular intervention and this is a second important standardized metric to assess MSU operations. Our MSU transported an average of 0.119 MT/day and meta-analysis revealed a range from 0.019 to 0.270, with an average rate of 0.092 (95% CI 0.046–0.138). Rates of MT/day follow a similar pattern to tPA administration per day, and also increase with catchment size and population density. Zhao et al. and the BEST MSU trial had the highest rates at 0.270 and 0.168, and they served the largest cohorts of the seven studies included (4, 14). Kate et al. (13) covers rural Alberta, with a very low population density, and reported lower rates of MT/day at 0.057. The Alberta program represents an instance in which MT/day and MT/dispatch offer conflicting portrayals of MSU activity, as the more specific dispatch criteria required in a rural setting raise the MT/dispatch rate to the higher end of the spectrum (9.0% compared to the average of 2.0% and range of 0.9–9.0%) (13). Rural programs often have smaller populations and longer drive times, which increase the difficulty of administering time sensitive therapies such as tPA and endovascular thrombectomy.

Two metrics that can be useful for internal comparison to evaluate the developmental stage of a mobile stroke program are dispatches/day and transportations/dispatch. Over the duration of this study, our program averaged 8.5 dispatches/day, ranging as low as 3.9 in the beginning and plateauing around 12.0/day. Transportation/dispatch rate was 18.8% (95% CI: 17.0–20.7%) at our institution, which decreased over the period of the study. These metrics lack external validity because of variable dispatch and transport criteria. To illustrate, Grunwald et al. had the highest rate of transportation/dispatch but transported a much lower percentage of patients with a final cerebrovascular diagnosis (39.7%) than we have previously reported (59%) (7, 23).

Utilization rates have not been previously reported but may serve as a metric for MSU programs to ensure efficiency and optimal resource utilization. Determining the ideal number of physicians, ambulances, and other resources to devote to an MSU program, requires an awareness of the frequency with which these resources are being utilized. In population-dense environments, MSU programs may progress to a utilization threshold at which they are unable to attend stroke alerts. The PHANTOM-S cohort in Berlin reported an inability to deploy their mobile stroke team to 1,288 patients, because the MSU was already in operation (2). They have since added two new MSUs to the Berlin region, resulting in expanded availability and coverage (9). The average utilization rate for our MSU was 13.3%, with a peak quarter of 21.9%. We believe this indicates there is capacity remaining within the system. Our utilization rate remained relatively stable since the inception of our MSU, despite increasing dispatch/day. We recommend monitoring utilization rates to ensure efficiency in maturing mobile stroke programs. Utilization rates as a function of operational time of day could be an avenue for future research.

### Context

Currently benchmarks are utilized for multiple clinical outcomes for stroke, including “door to needle” time and “door to groin puncture” time. However, little attention has been given to MSU operational performance. The data for these metrics was abstracted from existing manuscripts but was not reported in this format. The data we report may provide a preliminary benchmark for new or inexperienced programs looking to improve MSU operational efficiency. Establishing these operational performance metrics will also increase transparency between institutions. A successful MSU utilizes significant healthcare resources, including a specialized ambulance equipped with a CT scanner and a comprehensive stroke team. The standardized reporting of these performance metrics may allow for better comparison of MSUs and their utilization across different centers, while providing valuable information to institutions attempting to maximize cost-effectiveness and minimize resource utilization.

This study has several limitations. Although the data we reported was collected prospectively, it was analyzed in a retrospective fashion. Many of the studies included in the meta-analysis were also retrospectively analyzed. Therefore, these studies are subject to the typical biases associated with retrospective studies including confounding and selection bias. This is consistent with the publications bias that we observed within the transportations/dispatch analysis. This could suggest that the data within the literature is reported by high utilizers, and this should be considered when interpreting the results of this study. BEST-MSU and Berlin were prospective, controlled trials with data that was largely concordant with the other included studies (8, 9). This systematic review and meta-analysis included a variety of institutions which varied in experience, acuity, volume, and patient population. Each of these differences increased the observed heterogeneity between each group. This significant degree of heterogeneity suggests variability of utilization metric reporting and study design within the current literature and argues for better standardization and reporting.

## Conclusion

In this retrospective single institution analysis and meta-analysis, we propose and benchmark the following metrics for MSU operations: tPA/dispatch, tPA/day, MT/dispatch, MT/day, and utilization rate. These metrics are useful for internal and external comparison for institutions with or considering developing mobile stroke programs.

## Data Availability Statement

The raw data supporting the conclusions of this article will be made available by the authors, without undue reservation.

## Ethics Statement

The studies involving human participants were reviewed and approved by University of Rochester Institutional Review Board. Written informed consent for participation was not required for this study in accordance with the national legislation and the institutional requirements.

## Author Contributions

NE and DS contributed to the project conception, design, data collection, meta-analysis, manuscript drafting, review, and submission. RR and SA contributed to the project data collection, analysis, and manuscript revisions. AK and CB contributed to critically reviewing and revising the manuscript. SP contributed by providing additional data from other medical centers and reviewing the manuscript. JB, DP, WP, and TM contributed to data analysis, reviewed the manuscript, and provided critical revisions. JG contributed to the project conception, provided additional data from other medical centers, and reviewed and critically revised the manuscript. TB contributed to the project conception and reviewed and critically revised the manuscript. MB contributed to the project conception, design, data collection, meta-analysis, manuscript drafting, review, submission, and overseer of project. All authors contributed to manuscript revision, read, and approved the submitted version.

## Conflict of Interest

The authors declare that the research was conducted in the absence of any commercial or financial relationships that could be construed as a potential conflict of interest.

## Publisher's Note

All claims expressed in this article are solely those of the authors and do not necessarily represent those of their affiliated organizations, or those of the publisher, the editors and the reviewers. Any product that may be evaluated in this article, or claim that may be made by its manufacturer, is not guaranteed or endorsed by the publisher.
