# Supplementary material for: Mobile Stroke Unit Operational Metrics: Institutional Experience, Systematic Review and Meta-Analysis
Source: Front Neurol. 2022 May 9;13:868051. doi: 10.3389/fneur.2022.868051 (PMC9124821; doi:10.3389/fneur.2022.868051)
Supplement: Supplementary file 1 [file Table_1.DOCX]

Supplementary Table 1: Assessment of Heterogeneity

| **Operational Metric** | **I^2^** | **Significance Level** |
| --- | --- | --- |
| tPA/dispatch | 92.0% | P < 0.001 |
| tPA/day | 99.4% | P < 0.001 |
| MT/dispatch | 67.3% | P = 0.005 |
| MT/day | 90.0% | P < 0.001 |
| Transportations/dispatch | 99.1% | P < 0.001 |

tPA, tissue plasminogen activator; MT, mechanical thrombectomy

Supplementary Table 2: Risk of Bias Assessment using Egger’s Test

| **Operational Metric** | **Intercept** | **95% CI** | **Significance Level** |
| --- | --- | --- | --- |
| tPA/dispatch | 4.1463 | -2.86 to 11.15 | P = 0.1976 |
| tPA/day | -5.7032 | -29.91 to 18.51 | P = 0.5948 |
| MT/dispatch | 2.8022 | -0.51 to 6.11 | P = 0.0817 |
| MT/day | 5.1209 | -5.82 to 16.06 | P = 0.2828 |
| Transportations/dispatch | 15.9757 | 5.50 to 26.45 | P = 0.0097 |

CI, Confidence Interval; tPA, tissue plasminogen activator; MT, mechanical thrombectomy
